# The effect of nighttime macronutrient choice and exercise training on resting metabolic rate, appetite, and body composition in overweight and obese men and women

**DOI:** 10.1186/1550-2783-9-S1-P6

**Published:** 2012-11-19

**Authors:** Wyatt R Eddy, AW Kinsey, TA Madzima, CJ Blay, DD Thomas, LB Panton, J Kim, Michael J Ormsbee

**Affiliations:** 1 The Department of Nutrition, Food, & Exercise Sciences. The Florida State University, Tallahassee, FL, 32306, USA

## Background

Nighttime eating is often associated with metabolic syndrome and poor body composition and these conditions may be influenced by the natural decline in metabolism that occurs during sleep. However, previous research indicates that protein consumption increases metabolic rate more than carbohydrates or fat, and therefore may attenuate this decline when consumed at night before bed. In addition, digestion and absorption kinetics of whey protein (WP) and casein protein (CP) may independently influence appetite and body composition. Therefore, altering the type of protein or macronutrient consumed late at night when starting an exercise training program may influence changes in resting metabolic rate (RMR), appetite (hunger, desire to eat, and satiety), and body composition. The purpose of this study was to compare the effects of isocaloric maltodextrin (PLA), WP and CP supplements when consumed immediately prior to nocturnal sleep when combined with four weeks of exercise training on RMR, appetite, and body composition.

## Methods

Fifty-nine sedentary, overweight and obese volunteers were recruited and had baseline measurements of RMR, body composition (DXA), and appetite questionnaires taken after an overnight fast (0600-0900 h). Forty-eight completed the four-week study protocol. The participants were randomly assigned to one of three groups: PLA (n= 14, men: 4, BMI= 34.4 ± 1.5, age= 28.1 ± 1.8 years), WP (n= 17, men: 3, BMI= 34.3 ± 1.3, age= 30.1 ± 1.6 years), CP (n=17, men: 3, BMI= 35.4 ± 1.3, age= 30.1 ± 1.6 years) in a double blind design. Participants were then instructed to consume their supplement at least two hours after dinner and no more than 30 minutes before bed each night for four weeks. All participants attended supervised exercise sessions (3x/week; 2 days of resistance exercise and 1 day of high-intensity cardiovascular exercise). A one-way ANOVA was performed to examine possible group differences at baseline and differences in change between groups. Two-way ANOVA with repeated measures was used to evaluate changes in dependent variables over time ([pre x post] x [PLA x WP x CP]). A Tukey test was used for post hoc comparisons. Values are reported as means ± SEM.

## Results

Eleven participants who completed baseline measurements failed to complete the four-week protocol and maintain satisfactory compliance with exercise and supplement intake (> 80% compliance). No significant group differences existed at baseline. There were no group x time interactions for RMR, hunger, satiety, desire to eat, fat mass, lean body mass, or weight (*P*< 0.05), although RMR displayed a trend towards significance with the PLA group decreasing by 74.3 ± 94.5 and WP and CP increasing by 235.73 ± 84.5 and 51.7 ± 79.4kcal/day, respectively (*P*=0.0559). Significant time effects were measured for satiety (pre: 31.5 ± 2.3, post: 40.6 ± 2.3, *P*< 0.008) and LBM (pre: 51.8 ± 0.1, post: 52.3 ± 0.1, *P*< 0.0001).

## Conclusions

Our data indicate protein type and macronutrient choice in the late evening may not influence changes in RMR, hunger, desire to eat, satiety, and body composition during the first four weeks of an exercise intervention in sedentary, overweight and obese individuals.

